# Robust production of recombinant phosphoproteins using cell-free protein synthesis

**DOI:** 10.1038/ncomms9168

**Published:** 2015-09-09

**Authors:** Javin P. Oza, Hans R. Aerni, Natasha L. Pirman, Karl W. Barber, Charlotte M. ter Haar, Svetlana Rogulina, Matthew B. Amrofell, Farren J. Isaacs, Jesse Rinehart, Michael C. Jewett

**Affiliations:** 1 Department of Chemical and Biological Engineering, Northwestern University, 2145 Sheridan Road, Evanston, Illinois 60208, USA; 2 Northwestern Institute on Complex Systems, Northwestern University, 2145 Sheridan Road, Evanston, Illinois 60208, USA; 3 Simpson Querrey Institute, Northwestern University, Chicago, Illinois 60611, USA; 4 Chemistry of Life Processes Institute, Northwestern University, 2145 Sheridan Road, Evanston, Illinois 60208, USA; 5 Department of Cellular and Molecular Physiology, Yale University, New Haven, Connecticut 06520, USA; 6 Systems Biology Institute, Yale University, West Haven, Connecticut 06516, USA; 7 Department of Biomedical Engineering, Northwestern University, 2145 Sheridan Road, Evanston, Illinois 60208, USA; 8 Department of Molecular, Cellular and Developmental Biology, Yale University, New Haven, Connecticut 06510, USA; 9 Interdisciplinary Biological Sciences Program, Northwestern University, 2145 Sheridan Road, Evanston, Illinois 60208, USA

## Abstract

Understanding the functional and structural consequences of site-specific protein phosphorylation has remained limited by our inability to produce phosphoproteins at high yields. Here we address this limitation by developing a cell-free protein synthesis (CFPS) platform that employs crude extracts from a genomically recoded strain of *Escherichia coli* for site-specific, co-translational incorporation of phosphoserine into proteins. We apply this system to the robust production of up to milligram quantities of human MEK1 kinase. Then, we recapitulate a physiological signalling cascade *in vitro* to evaluate the contributions of site-specific phosphorylation of mono- and doubly phosphorylated forms on MEK1 activity. We discover that only one phosphorylation event is necessary and sufficient for MEK1 activity. Our work sets the stage for using CFPS as a rapid high-throughput technology platform for direct expression of programmable phosphoproteins containing multiple phosphorylated residues. This work will facilitate study of phosphorylation-dependent structure–function relationships, kinase signalling networks and kinase inhibitor drugs.

## Abstract (web summary)

The inability to produce recombinant phosphoproteins has hindered research into their structure and function. Here the authors develop a cell-free protein synthesis platform to site-specifically incorporate phosphoserine into proteins at high yields, and recapitulate a MEK1 kinase signalling cascade.

## Graphical abstract

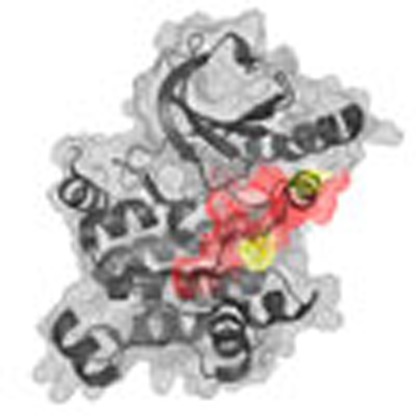

Phosphorylation, the reversible attachment of phosphate groups to proteins, is one of the most important post-translational modifications (PTMs) in nature, serving as a mechanism for enormous diversification in the function and molecular recognition of proteins^1^,^2^. However, understanding the role of site-specific phosphorylation events remains a significant challenge due to technological limitations. Specifically, the inability to produce phosphoproteins with defined phosphorylation status at high purity and yield has restricted our capacity to elucidate the phosphorylation ‘code’ within the human phosphoproteome, probe complex signalling networks and develop novel phosphoprotein-based therapeutics. Moreover, most phosphoproteins isolated from cells and tissues are complex heterogeneous mixtures of different chemical structures owing to their mode of biosynthesis, subcellular distribution and diversity of PTMs. Furthermore, extensive crosstalk between kinase and phosphatase networks *in vivo* expands the spatio-temporal diversity of PTMs within the cell^3^, confounding genetic approaches to decode the relationship between a specific phosphorylation event and its biological function. Thus, a simple technology capable of producing useful quantities of proteins featuring multiple, user-specified phosphorylation sites for biochemical and structural biology studies would be transformative.

Various methods have been developed to manufacture and study phosphorylated proteins^4^. State-of-the-art approaches that mimic phosphorylation by replacing a target site with an aspartate/glutamate residue or chemical analogue are powerful^5^, yet are limited by their inability to match the charge density of the phosphate group. Alternatively, kinases known to phosphorylate a target site of interest have been employed *in vitro*. However, *in vitro* kinase phosphorylation often lacks site specificity^6^. In a different strategy, semisynthetic approaches can use native chemical ligation, which has proven useful but is limited to sites near the protein termini^7^,^8^. More recently, pioneering efforts have expanded the genetic code of *Escherichia coli* for site-specific incorporation of L-phosphoserine (Sep) into proteins using amber codon suppression^9^,^10^,^11^,^12^,^13^. While these systems have been used for producing proteins harbouring Sep *in vivo*, protein expression yields remain low (∼ 1.1 μg ml^−1^ green fluorescent protein (GFP))^11^.

Cell-free protein synthesis (CFPS) systems have emerged to help meet increasing demands for simple and efficient protein expression, with *E. coli*-based CFPS systems now exceeding grams of protein per liter reaction volume for proteins^14^. Notably, the open nature of these reactions allows the user to directly influence the biochemical systems of interest. As a result, new components can be added/synthesized and maintained at precise concentrations, circumventing limitations in cellular uptake of non-standard amino acids (nsAAs). Furthermore, cell-free systems bypass limits imposed by the fitness of the organism. This enables the use of cytotoxic nsAAs, the production of cytotoxic proteins and removes constraints arising from toxic orthogonal translation system (OTS) components^11^. Recent works highlight the ability of CFPS to synthesize complex human proteins in high throughput^14^, even some harbouring site-specifically incorporated nsAAs^15^,^16^,^17^,^18^,^19^,^20^,^21^,^22^,^23^,^24^, which is the focus of this work.

We developed a CFPS system that can substantially improve throughput and yields of phosphoprotein synthesis (Fig. 1a). This approach comprised three important features. First, highly active S30 crude extracts were generated from a genomically recoded release factor 1-deficient *E. coli* strain (*E. coli* C321.ΔA)^25^ that also lacks the Sep-specific phosphatase *SerB*. Second, an improved Sep-OTS^9^ was expressed during the growth of the recoded *E. coli* strain to enrich the lysate with the OTS components (that is, phosphoseryl-tRNA synthetase, tRNA^Sep^ and EF-Sep) necessary for phosphoprotein synthesis (Fig. 1b). The Sep-OTS was improved from previous efforts^12^,^26^ by combining the OTS components onto one vector, and by increasing the tRNA^Sep^ gene copy number from 1 to 5 (ref. 12). Third, these engineered components were integrated into a CFPS platform that has been previously shown to mimic the *E. coli* cytoplasmic environment^27^,^28^. We used this platform to activate long-lived and efficient cell-free phosphoprotein synthesis.

## Results

### Cell-free phosphoprotein synthesis of sfGFP

**Figure 1:**
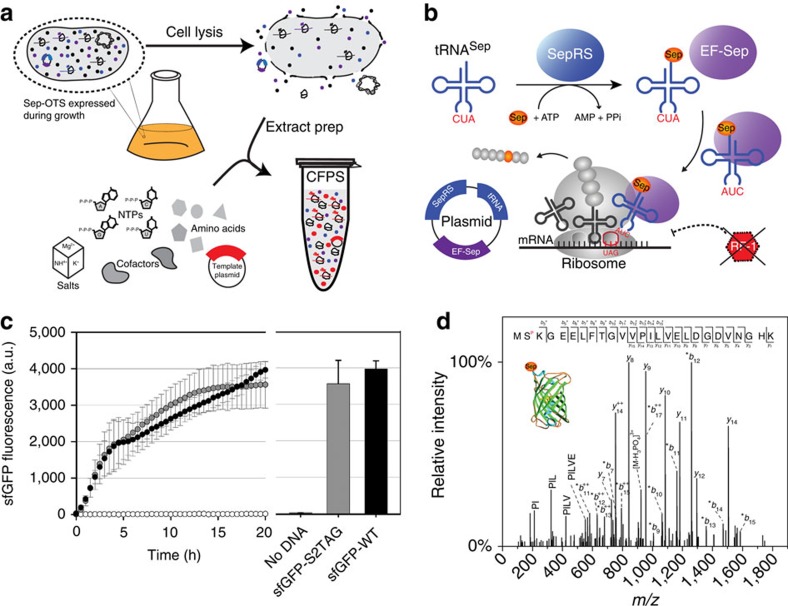
CFPS platform with an expanded genetic code for the production of phosphoproteins. (**a**) Schematic of the production and utilization of an S30 crude extract containing the Sep-OTS for phosphoprotein biosynthesis. The plasmid-based Sep-OTS is induced during cell growth in the presence of Sep supplemented to the culture media. Cells expressing the Sep-OTS are then collected, lysed and processed to generate S30 extracts. CFPS reactions are supplemented with nucleoside triphosphates (NTPs), amino acids, T7 RNA polymerase and template plasmid DNA to direct the transcription and translation of a desired phosphoprotein product. (**b**) Schematic of the Sep-OTS. tRNA^Sep^ is aminoacylated with Sep by SepRS. EF-Sep then delivers Sep-tRNA^Sep^ to the ribosome. Site-specific incorporation of Sep at UAG (amber codon) is directed via the CUA anticodon of tRNA^Sep^. (**c**) Time-course and endpoint analysis of sfGFP-S2TAG with sfGFP-S2TAG DNA template added (dark grey) and as a control with no template DNA added (white). Expression of wild-type sfGFP-S2S (black) in the presence of the Sep-OTS. Error bars represent s.d. from three independent samples. (**d**) Annotated tandem mass spectrum from sfGFP-S2TAG confirming the site-specific incorporation of Sep at position S2. Doubly charged ions and fragments that have lost ammonia are marked by ++ and * respectively.

Our goal was to develop a CFPS platform capable of rapidly synthesizing an active human kinase. To accomplish this, we first optimized conditions that enabled site-specific incorporation of Sep into the superfolder GFP (sfGFP) reporter protein at a single in-frame amber codon at position 2 (sfGFP-S2TAG). Notably, the *sfGFP* gene sequence also encoded a C-terminal Strep-tag to enable purification and proteomics analysis. To assess phosphoprotein production in CFPS, 15 μl scale batch reactions were conducted for 20 h at 30 °C using the PANOx-SP system^27^,^28^. Combined transcription and translation in the CFPS reaction were driven by either the wild-type sfGFP or the sfGFP-S2TAG DNA template. Negative controls lacking DNA templates were also conducted. Using this approach, we synthesized 686±48 μg ml^−1^ of wild-type sfGFP and 567±37 μg ml^−1^ sfGFP-S2TAG in batch CFPS reactions that used extracts containing the overexpressed Sep-OTS (Fig. 1c, Table 1; mean±s.d.; *n*=4).

Affinity-purified sfGFP was analysed by SDS–polyacrylamide gel electrophoresis (PAGE) to confirm production of full-length protein (Supplementary Fig. 1). We then digested the affinity-purified sfGFP with trypsin and confirmed incorporation of Sep at sfGFP-S2TAG by mass spectrometry (MS) using label-free shotgun proteomics. A representative tandem MS spectrum from a N-terminal tryptic peptide of sfGFP reporting incorporation of Sep at position S2 of sfGFP is provided in Fig. 1d. To assess the purity of modified sfGFP, we performed label-free quantitation using two strategies: spectral counting and MS1 intensity-based quantitation (Supplementary Tables 1–3, Supplementary Figs 2 and 3). Both approaches confirmed that the majority of sfGFP expressed with the Sep-OTS system contained Sep incorporated at position 2 of sfGFP-S2TAG. Near-cognate suppression via incorporation of amino acids Q, Y and K was responsible for read-through of the TAG codon as we had observed in our previous studies^12^,^25^ (Supplementary Table 1). We also found low levels of glycine read-through of the TAG codon. However, the incorporation of these natural amino acids was dramatically reduced in the presence of the Sep-OTS (Supplementary Figs 2 and 3). Last, the high sensitivity of our MS assay allowed us to detect direct evidence for ribosomal skipping at the TAG codon (Supplementary Tables 1–3). Relative intensities of peptides harbouring ribosomal skipping were 12.2 and 2.1% in the absence or presence of the Sep-OTS, respectively (Supplementary Table 3 and Supplementary Fig. 3).

Prior studies have suggested that the efficiency of amber codon suppression may be position dependent^22^,^29^,^30^. In other words, the position in which the nsAA is incorporated affects protein synthesis yields and incorporation efficiency. To study this effect, we further examined Sep incorporation in sfGFP at position E17. While sfGFP-E17TAG yields remained high at 516±9 μg ml^−1^ (Table 1), efficiency of Sep incorporation as determined by MS1 intensity-based quantitation was lower, ∼20% for sfGFP-E17TAG versus ∼90% for sfGFP-S2TAG (Supplementary Table 3). Similar to our results with S2, near-cognate suppression via incorporation of amino acids Q, Y, K and G was also observed at position 17 (Supplementary Table 1, Supplementary Fig. 4). Our results provide evidence for the positional dependence of nonsense suppression efficiency.

Since we intended to study a human kinase with two Sep residues (see below), we also studied site-specific phosphorylation into sfGFP at positions S2 and E17. As compared with wild-type sfGFP expression, yields were lower when synthesizing sfGFP-S2TAG/E17TAG (289±21 μg ml^−1^; Table 1). Unfortunately, efforts to detect the doubly phosphorylated sfGFP by MS failed due to technical limitations. Specifically, addition of multiple phosphorylated residues into peptides reduces their ionization efficiency. In addition, we speculate that the addition of two negative charges into the reporter peptide shifts the charge state of the peptide to a singly charged peptide with an *m/z* ratio outside of our detection window. To verify Sep incorporation in doubly phosphorylated sfGFP and validate both mono-phosphorylated forms, we used a Phos-tag-mediated mobility shift assay that has been previously described^31^,^32^ (Supplementary Fig. 5). The Phos-tag reagent binds phosphorylated proteins and causes slower migration of phosphorylated proteins during SDS–PAGE. Using the Phos-tag method, we observed distinct signature shifts for phosphoserine-containing fractions of S2TAG, E17TAG and S2TAG/E17TAG sfGFP preparations compared with the unmodified, wild-type sfGFP (Supplementary Fig. 5). Overall, Phos-tag and MS data validated a novel CFPS platform that permits biosynthesis of proteins harbouring site-specifically incorporated Sep residues.

### Cell-free phosphoprotein synthesis of MEK1 kinase

**Figure 2:**
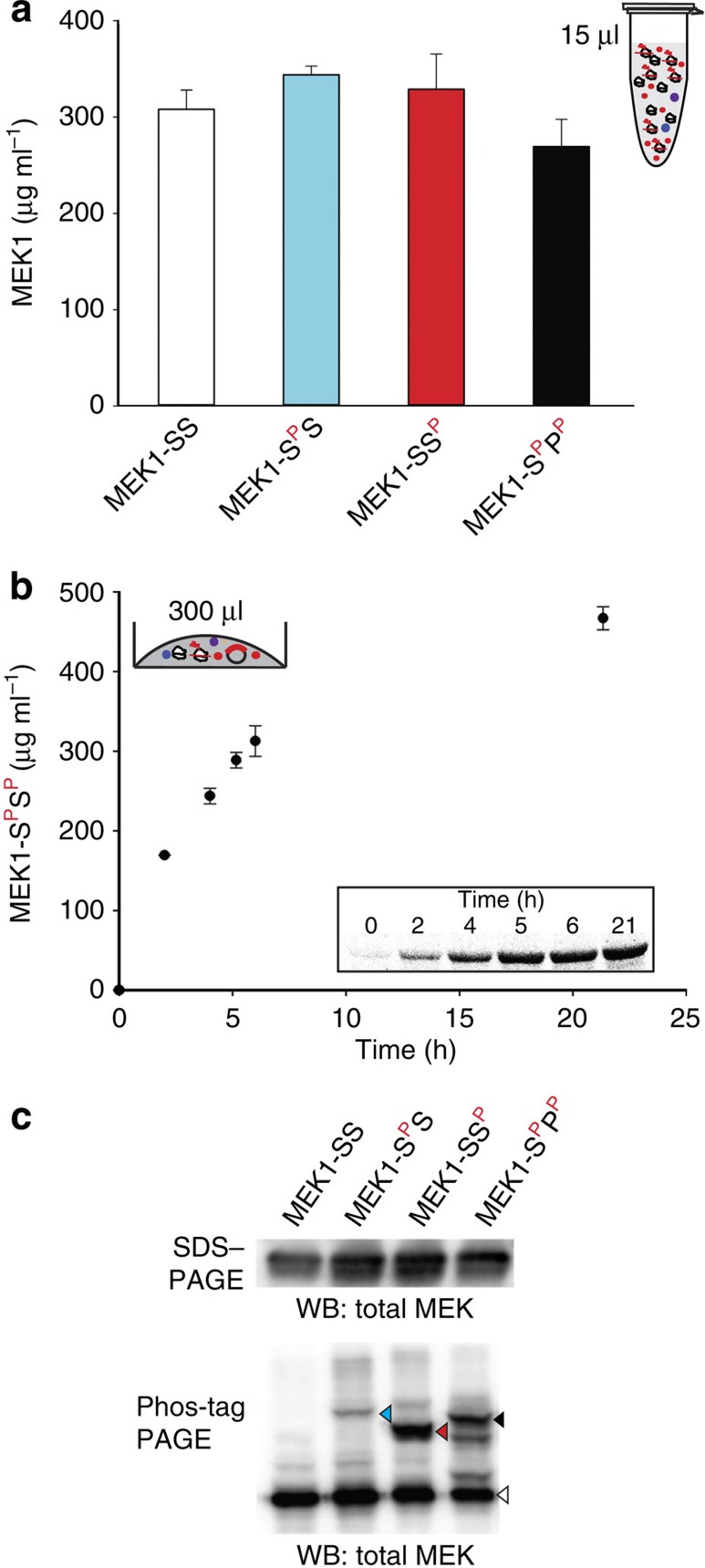
*In vitro* synthesis of phosphorylated MEK1 variants. (**a**) Volumetric yields of phosphorylated MEK1 variants MEK1-SS, MEK1-S^P^S, MEK1-SS^P^ and MEK1-S^P^S^P^ produced in 15 μl batch reactions. (**b**) Time-course plot showing the biosynthesis of MEK1-S^P^S^P^ in 300 μl batch reactions. The inset shows a representative autoradiogram of MEK1-S^P^S^P^ being synthesized over time. (**c**) Quantitation of total MEK1 production and phosphoprotein production by western blot analysis on SDS–PAGE and Phos-tag gels. The Phos-tag western blot shows characteristic shifted bands for the phosphorylated MEK1 variants MEK1-S^P^S, MEK1-SS^P^ and MEK1-S^P^S^P^. These bands are absent in the wild-type MEK1-SS control sample. Error bars represent s.d. from three independent samples. *N*=3 for all western blot and gel analysis.

We next demonstrated the utility of our method by applying the platform for the *in vitro* synthesis of highly active doubly phosphorylated human MEK1 kinase (mitogen-activated ERK activating kinase 1). In previous works, bacterial expression of soluble MEK1 *in vivo* has required fusion partners, such as the maltose binding protein^9^ or glutathione *S*-transferase^33^ fusion tags. Since CFPS systems have shown benefits for expressing mammalian proteins in soluble form as compared with *in vivo* expression platforms^34^, we attempted to express MEK1 without fusion partners. Wild-type MEK1 with serines at positions S218 and S222 (MEK1-SS) and doubly phosphorylated MEK1 with phosphoserines at the same positions (MEK1-S^P^S^P^) were produced in 20-h CFPS batch reactions at 30 °C (Fig. 2a). A unique feature of our technology for co-translational incorporation of Sep is the ability to produce mono-phosphorylated forms of kinases without having to add alanine mutations to suppress second site phosphorylation events. We therefore also synthesized MEK1 variants phosphorylated at either S218 (MEK1-S^P^S) or S222 (MEK1-SS^P^) having the native activation loop amino-acid sequence to determine if each site individually could activate the kinase alone. The total MEK1 expression yields for each variant were quantified by ^14^C-leucine incorporation. We observed synthesis of 308±19 μg ml^−1^ MEK1-SS, 343±9 μg ml^−1^ MEK1-S^P^S, 328±36 μg ml^−1^ MEK1-SS^P^ and 269±28 μg ml^−1^ of MEK1-S^P^S^P^ (Table 1, Fig. 2a). These volumetric yields (g l^−1^) exceeded previously reported in cell produced MEK1 by >1,000-fold (Table 1).

While phosphoprotein production in 15 μl batch reactions provides throughput, we next set out to demonstrate the potential for scale-up, noting that several previous works have demonstrated the ability to scale CFPS to the litre scale^35^,^36^. For example, Yin *et al*.^36^ used the open reaction environment of CFPS to produce 300 μg ml^−1^ aglycosylated trastuzumab in reactions ranging from 60 μl to 4 l. By increasing the batch reaction scale more than 10-fold to 300 μl reactions in a 24-well flat-bottom plate, we observed production of 467±15 μg ml^−1^ MEK1-S^P^S^P^ (Fig. 2b). The increase in productivity from our 15 μl reactions is consistent with previous reports^37^,^38^, which have noted that increasing the surface area-to-volume ratio can increase CFPS yields. We pooled together eight CFPS reactions to make 1.05±0.12 mg of total MEK1-S^P^S^P^ as determined by radioactive incorporation (Fig. 2b). The capacity to manufacture milligram quantities of site-specifically phosphorylated proteins demonstrates that our approach is not restricted to microgram quantities for analytical purposes.

Having successfully expressed different forms of MEK1, we next characterized the protein products in several ways. First, we validated the production of full-length MEK1 by western blot analysis using an antibody specific for the C-terminal His tag (Fig. 2c; full gels available in Supplementary Fig. 6). As expected, full-length MEK1, MEK1-S^P^S, MEK1-SS^P^ and MEK1-S^P^S^P^ were produced when the DNA template contained site-specific in-frame amber codons. Second, we confirmed MEK1-S^P^S^P^ production by western blot using a phosphospecific antibody for MEK1 (Supplementary Fig. 7). Third, we validated the presence of Sep using the Phos-Tag gel-shift assay, because similar to doubly phosphorylated GFP, we were unable to detect the MEK1-S^P^S^P^ peptide by MS for technical reasons. The gel shifts for MEK1-S^P^S, MEK1-SS^P^ and MEK1-S^P^S^P^ were distinct, and as noted by others^39^, the doubly phosphorylated MEK1 protein counter-intuitively produced a characteristic shift that was intermediate when compared with the mono-phosphorylated MEK1 variants (Fig. 2c), an observation that is also consistent with the phosphorylated sfGFP (Supplementary Fig. 5). Our Phos-tag data provided clear demonstration of single- and two-site phosphorylation into MEK1. While our data suggest that the product is not completely pure (that is, there are some non-phosphorylated species), we believe that continued developments to build a more efficient Sep-OTS^40^,^41^ or removal or phosphatases from the extract will provide notable improvements in purity as seen for other OTSs. We then used our platform to elucidate sequence-function relationships for MEK1.

### Functional effects of site-specific phosphorylation in MEK1

**Figure 3:**
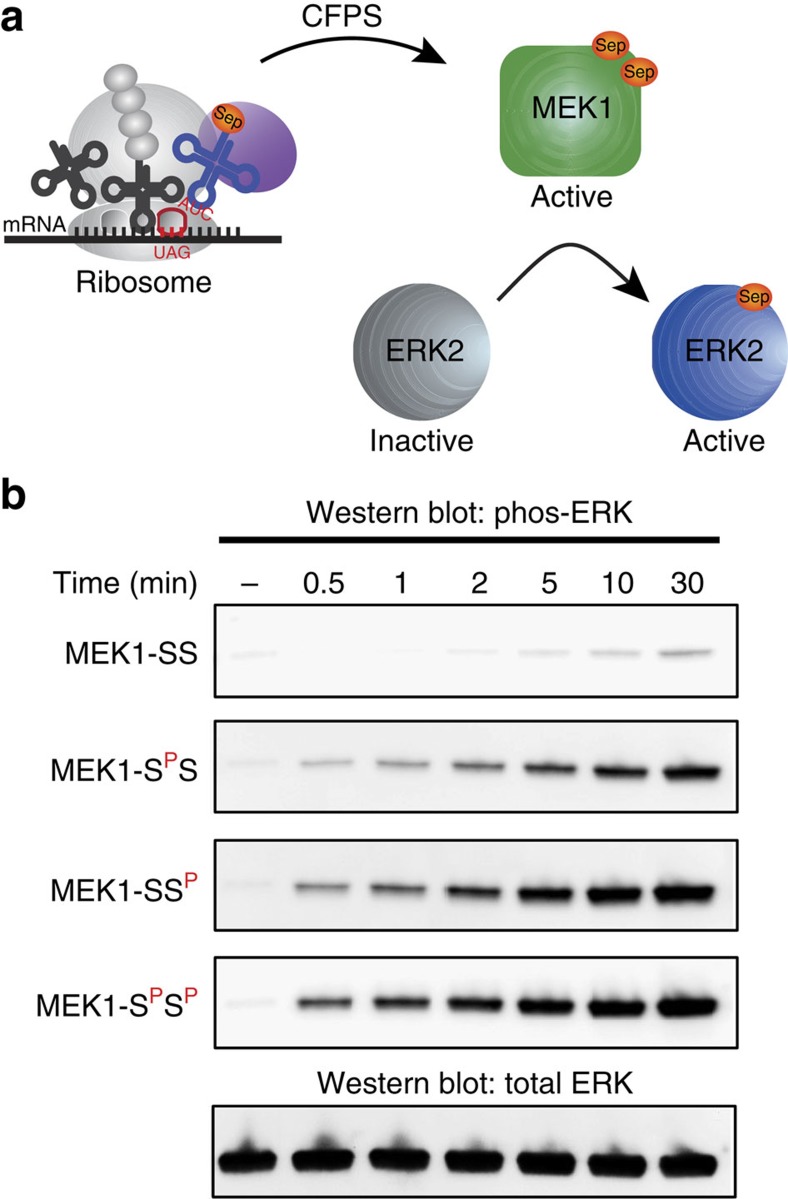
Assessment of phosphorylated MEK1 activity. (**a**) Schematic showing the production of MEK1 variants using CFPS and subsequent activity testing with an *in vitro* kinase assay using the native MEK1 substrate ERK2. (**b**) *In vitro* MEK1 kinase activity was measured at 0.5, 1, 2, 5, 10 and 30 min time points using kinase dead ERK2 as a substrate. Background ERK2 phosphorylation was determined in the absence of MEK1 protein (far left lane). Representative western blots of phosphorylated ERK (Phos-ERK) confirm the increased activity of phosphorylated MEK1 kinase variants in reactions carried out with equal amounts of ERK substrate. Total ERK was assayed by western blot analysis with an anti-His antibody. *N*=3 for all kinase assays and western blot analysis.

MEK1 plays an important role in cellular signal transduction through the human MAP kinase cascade responsible for driving cellular proliferation and differentiation^42^. Within the cascade, the doubly phosphorylated MEK1 phosphorylates the extracellular-signal-regulated kinases ERK1 and ERK2 (Fig. 3a). We therefore examined the enzymatic activity of cell-free synthesized mono- and doubly phosphorylated MEK1 variants towards ERK2 with an *in vitro* kinase cascade assay. To evaluate kinase activities, we incubated the CFPS reaction products MEK1-SS, MEK1-S^P^S, MEK1-SS^P^ or MEK1-S^P^S^P^ with a full-length ERK2 K54R variant with greatly reduced autophosphorylation activity. Western blot analysis with an anti-phos-ERK antibody showed that the doubly phosphorylated MEK1 could robustly phosphorylate ERK2 *in vitro* as expected (Fig. 3b; full gels available in Supplementary Fig. 7). Uniquely, our platform also enabled us to test the functional role of mono-phosphorylated forms on MEK1 activity. We discovered that phosphorylation at either S218 or S222 is necessary and sufficient for MEK1 kinase activity (Fig. 3b). To our knowledge, this is the first demonstration that three different forms of MEK1, with native phosphoserine, produce active kinase. It is also the first demonstration of active MEK1 synthesis by bacterial CFPS without the need for fusion proteins to enable soluble protein expression. Obviating fusion proteins during the production of human proteins in CFPS systems as demonstrated here will provide new opportunities to study the kinase without confounding effects of the solubility protein partner. The flexibility and utility of the CFPS system is shown by the rapid, robust and scalable production of the active phosphorylated MEK1-S^P^S^P^ for biochemical and biophysical characterization.

## Discussion

By expanding the genetic code in the CFPS environment our approach has shown the ability to rapidly produce (<24 h) and test an active human phosphoprotein in high yields. The capacity to utilize chemically synthesized linear DNA libraries^14^ in this platform will enable high-throughput synthesis of phosphoproteins, markedly increasing the speed and resolution at which we can define, manipulate and study phosphorylation-induced effects on protein structure and function. Indeed, our cell-free approach can measure and study the contributions of site-specific phosphorylation to understand how phosphoproteins perform their critical and versatile roles in cellular regulation. Looking forward, the CFPS technology described herein for the direct synthesis and interrogation of phosphoproteins sets the stage for new technological paradigms to (i) understand the human phosphoproteome, (ii) develop novel arrays of disease-related phosphorylated proteins and (iii) identify novel small molecule inhibitors with therapeutic potential. Improvements to the Sep-OTS for enhanced incorporation efficiency will open the way to even broader applications.

## Methods

### Cell-free protein synthesis extract preparation

*E. coli* C321.ΔA cells^25^ harbouring the Sep-OTS system were grown in 2 × YTPG media^28^ supplemented with 2 mM L-phosphoserine at 30 °C. The SepOTS system was induced at an OD_600_ of 0.6 with 1 mM isopropyl-β-D-thiogalactoside (IPTG) and cells were grown to a final OD_600_ of 3.0 that represents the middle of the exponential growth phase. Cultures were then collected on ice and cells were pelleted by centrifugation performed for 15 min at 5,000 relative centrifugal force (RCF) and 4 °C. Cell pellets were then washed three times with cold S30 buffer (10 mM Tris-acetate pH 8.2, 14 mM magnesium acetate, 60 mM potassium acetate and 1 mM dithiothreitol). After final wash and centrifugation, the pelleted wet cells were weighed, flash frozen in liquid nitrogen and stored at −80 °C. To make cell extract, the thawed cells were suspended in 0.8 ml of S30 buffer per 1 g of wet cell mass and processed as reported by Kwon and Jewett^43^.

### Cell-free protein synthesis reactions

CFPS reactions were performed under conditions similar to the PANOx-SP system^27^,^43^. Batch reactions were conducted with a final reaction volume of 15 μl in 600-μl microfuge tubes. Reactions were performed either in duplicate or triplicate, with duplicate measurements for each condition tested. Reactions in 96-well plates (Costar 3694; Corning Incorporated, Corning, NY) were performed with a final reaction volume of 30 μl. All reactions contained 30% v/v cell extract supplemented with 1.2 mM ATP, 0.85 mM each of GTP, UTP and CTP; 34.0 μg ml^−1^ folinic acid; 170.0 μg ml^−1^ of *E. coli* tRNA mixture; 13.3 μg ml^−1^ plasmid; 100 μg ml^−1^ T7 RNA polymerase; 2 mM each of 20 standard amino acids; 0.33 mM NAD; 0.27 mM coenzyme-A (CoA); 1.5 mM spermidine; 1 mM putrescine; 4 mM sodium oxalate; 130 mM potassium glutamate; 10 mM ammonium glutamate; 12 mM magnesium glutamate; and 33 mM phosphoenolpyruvate. Reactions were conducted for 20 h at 30 °C in an incubator without shaking.

CFPS reactions were scaled to reaction volumes of 300 μl in untreated flat-bottom 24-well plates (Model 353226; BD Biosciences, San Jose, CA, USA). Eight 300-μl reactions were run in parallel for the production of milligram quantities of MEK1-S^P^S^P^ resulting in a total reaction volume of 2.4 ml. Remaining wells around the perimeter of the plate were filled with water for internal humidification, which resulted in reduced sample evaporation. Reactions in 24-well plates were conducted at 30 °C while shaking at 300 r.p.m. in a ThermoMixer (Eppendorf, Mississauga, Ontario). Full details for cell growth, cell harvest, and CFPS extract preparation are provided in the Supplementary Information.

### sfGFP western blot analysis

For western analysis of sfGFP, 100 ng of total sfGFP samples were loaded on either 4–15% acrylamide gels (Bio-Rad) or on a handcast 10-well 12% acrylamide gel containing 25 μM Phos-tag acrylamide. Transferred PVDF membranes were blotted with 1:545 Anti-GFP (Invitrogen, Mouse Anti-GFP 33-2,600) followed by 1:10,000 DAM-HRP. Signal was detected by enhanced chemiluminescence (Bio-Rad) imaged on a ChemiDoc XRS+CCD camera. Densitometry was performed using the Bio-Rad Image Lab software.

### MEK1 western blot analysis

Purified MEK1 protein was separated by SDS–PAGE on a 4–15% gradient polyacrylamide gel (Bio-Rad) under reducing and denaturing conditions, and transferred to PVDF membranes. Membranes were blotted with either 1:2,500 Anti-His (6x-His Epitope TAG, PA1-983B, Thermo Fisher Scientific) for total MEK1 or 1:1,000 Anti-MEK1-S^P^S^P^ (Phospho-MEK1 1/2 (Ser217/221), 9154, Cell Signaling Technology), followed by 1:10,000 DAR-HRP. Signal was detected by enhanced chemiluminescence (Bio-Rad) imaged on a ChemiDoc XRS+CCD camera. Densitometry was performed using the Bio-Rad Image Lab software.

### MEK1 kinase activity assay

Kinase activity of purified full-length MEK1 (S218/S222, S218/S^P^222, S^P^218/S222 or S^P^218/S^P^222) was evaluated by measuring ERK2 phosphorylation. Approximately, 1.0 μM MEK1 variants were pre-incubated in kinase activity buffer (50 mM Tris-HCl (pH=7.4), 150 mM NaCl, 1 mM dithiothreitol, 20% glycerol, 10 mM MgCl_2_ and 1 mM ATP) at 30 **°**C for 5 min, then 2.5 μM ERK2 substrate was added and the reaction was allowed to proceed at 30 °C. Aliquots of 7.5 μl were removed at 0, 0.5, 1, 2, 5, 10 and 30 min and quenched with 7.5 μl of 2 × Laemmli sample buffer, then heated to 55 °C for 5 min. A negative control was run with only ERK2 substrate for 30 min and quenched in the same manner as kinase-containing samples. The quenched reactions were run on 4–15% acrylamide SDS–PAGE gels and transferred to a PVDF membrane. Membranes were blotted with either 1:1,000 Anti-Phos-Erk antibody (Phospho p44/42 MAPK (Erk1/2) (Thr 202/Tyr204), 9101, Cell Signaling Technology) and 1:2,500 Anti-His antibody (6x-His Epitope TAG, PA1-983B, Thermo Fisher Scientific) for total MEK1, followed by 1:10,000 DAR-HRP. Signal was detected by enhanced chemiluminescence on a Bio-Rad ChemiDoc equipped with XRS+CCD camera. The kinase activity assay was run in triplicate using the same purified preparation of MEK1 kinase and K54R ERK2.

### Mass spectrometry and bioinformatics

MS procedures and sample preparation details are given in the Supplementary Methods.

## Additional information

**How to cite this article:** Oza, J. P. *et al*. Robust production of recombinant phosphoproteins using cell-free protein synthesis. *Nat. Commun.* 6:8168 doi: 10.1038/ncomms9168 (2015).

## Supplementary Material

Supplementary Information Supplementary Figures 1-8, Supplementary Tables 1-4, Supplementary Methods and Supplementary References

## Figures and Tables

**Table 1:** Comparison of CFPS titres in this study relative to previous works.

Protein	Yield (μg ml^−1^)		No. of TAG	Position	% Yield of WT	Strain	Reference
sfGFP	686	±48	0	0	WT	*E. coli* C321.ΔA	This work
sfGFP	567	±37	1	S2	83% of WT	*E. coli* C321.ΔA	This work
sfGFP	516	±9	1	E17	75% of WT	*E. coli* C321.ΔA	This work
sfGFP	289	±21	2	S2, E17	42% of WT	*E. coli* C321.ΔA	This work
MEK1	308	±20	0	0	WT	*E. coli* C321.ΔA	This work
MEK1	343	±9	1	S218	100% of WT	*E. coli* C321.ΔA	This work
MEK1	328	±36	1	S222	100% of WT	*E. coli* C321.ΔA	This work
MEK1	269	±28	2	S218, S222	87% of WT	*E. coli* C321.ΔA	This work
GFP	1.1	NA	1	E17	3.5% of WT	EcAR7	11
MEK1	0.001	NA	2	S218, S222	<4% of WT	BL21	9

NA, not applicable; WT, wild type.
