# Supplementary material for: Robust production of recombinant phosphoproteins using cell-free protein synthesis
Source: Nat Commun. 2015 Sep 9;6:8168. doi: 10.1038/ncomms9168 (PMC4566161; doi:10.1038/ncomms9168)
Supplement: Supplementary Information — Supplementary Figures 1-8, Supplementary Tables 1-4, Supplementary Methods and Supplementary References [file ncomms9168-s1.pdf]

Supplementary Figures

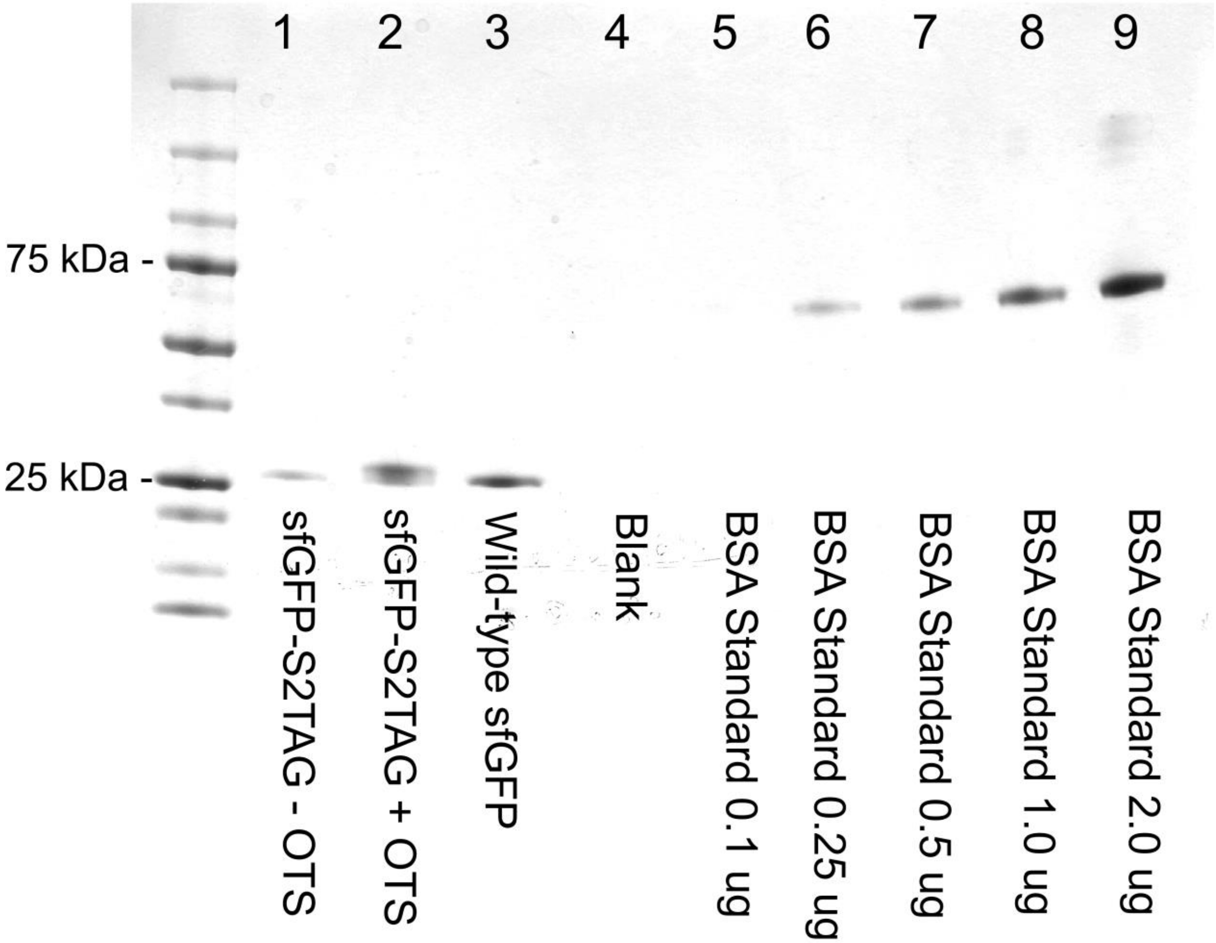

Supplementary Figure 1. SDS-PAGE gel of sfGFP produced by CFPS confirms production of full-length protein. Proteins from CFPS reactions were strep-tag affinity purified, separated by SDS-PAGE and visualized by Coomassie blue staining. sfGFP-S2TAG produced by CFPS in the absence and presence of the Sep-OTS (lanes 1-2) and wild-type sfGFP (lane 3) are the same molecular weight. Production of low-level sfGFP in absence of the Sep-OTS due to near-cognate misincorporation of amino acids also produces full-length protein (lane 1). BSA standards (0.1, 0.25, 0.5, 1.0 and 2.0 µg) were analyzed on the same gel to assist with the quantitation of sfGFP (lanes 5-9).

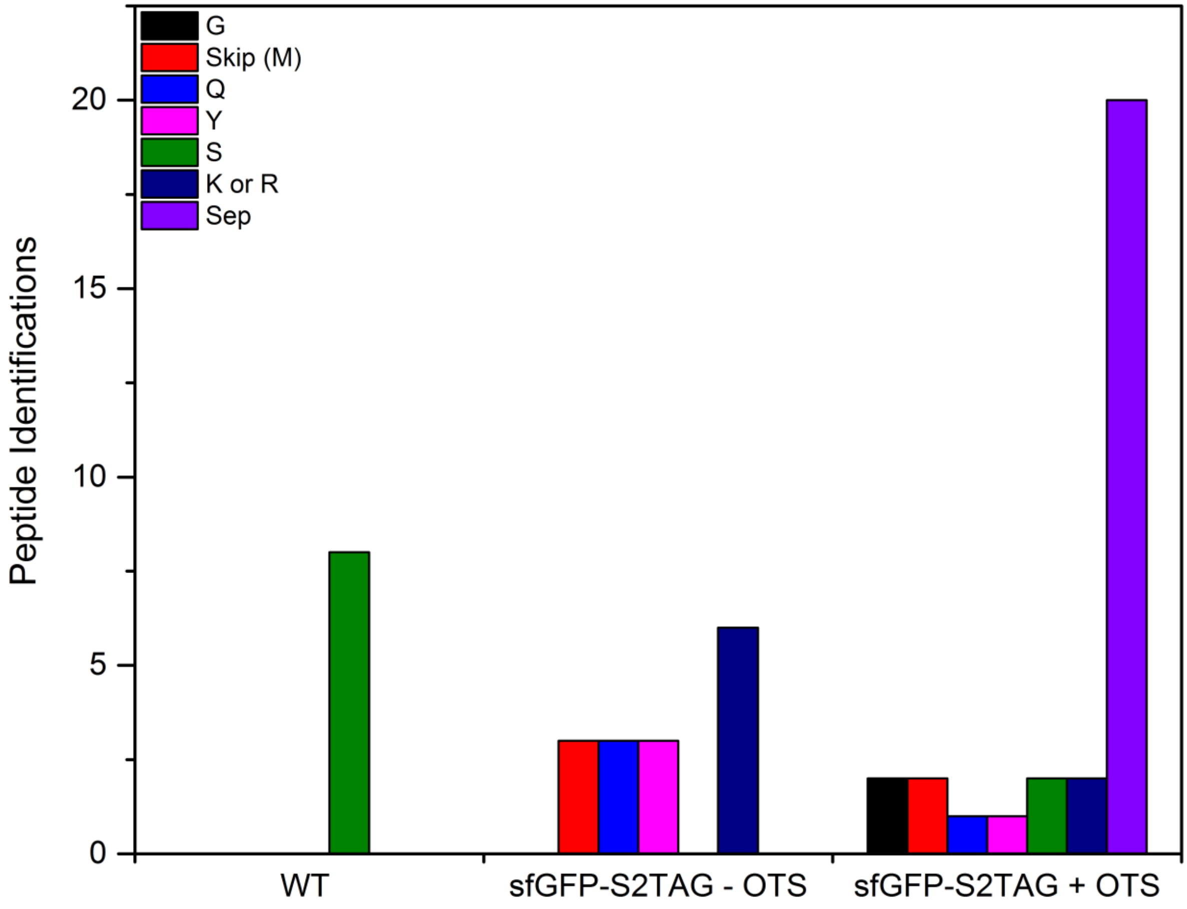

Supplementary Figure 2. Quantitation of Sep incorporation at position S2 of sfGFP-S2TAG by label-free proteomics using spectral counting. For spectral counting, the number of reported peptide identifications for peptides reporting position S2 of sfGFP was recorded (see Supplementary Table 2). The number of identified spectra reporting the incorporation of each amino acids at position S2 of sfGFP is plotted. The number of Sep containing peptides dramatically increases in presence of the Sep-OTS.

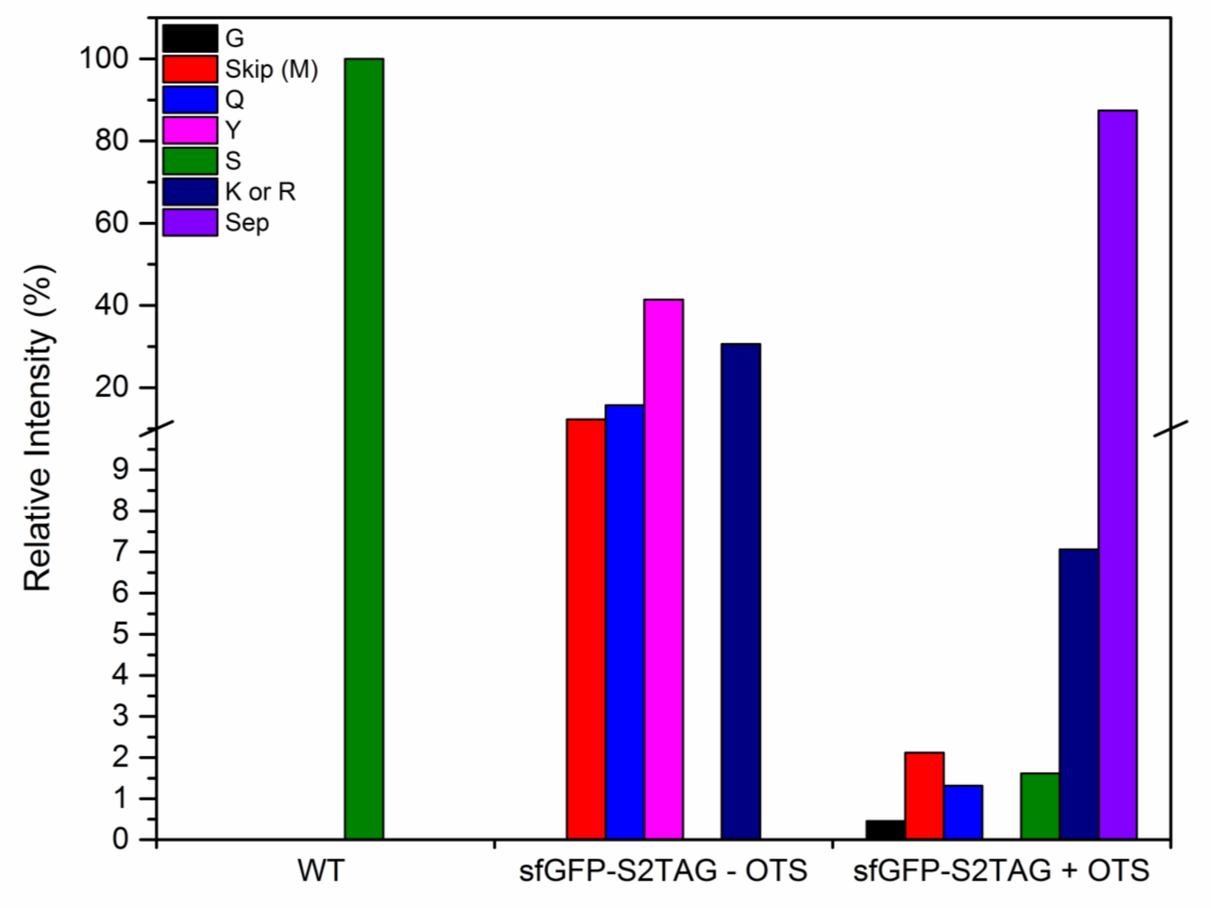

Supplementary Figure 3. Label-free quantitation of Sep incorporation into sfGFP at position S2. The normalized MS1 intensity of peptides reporting incorporation of amino acids are plotted as a percentage of the total ion intensity of peptides reporting incorporation of amino acids at position S2 of sfGFP (see Supplementary Table 3). Note the y-axis break.

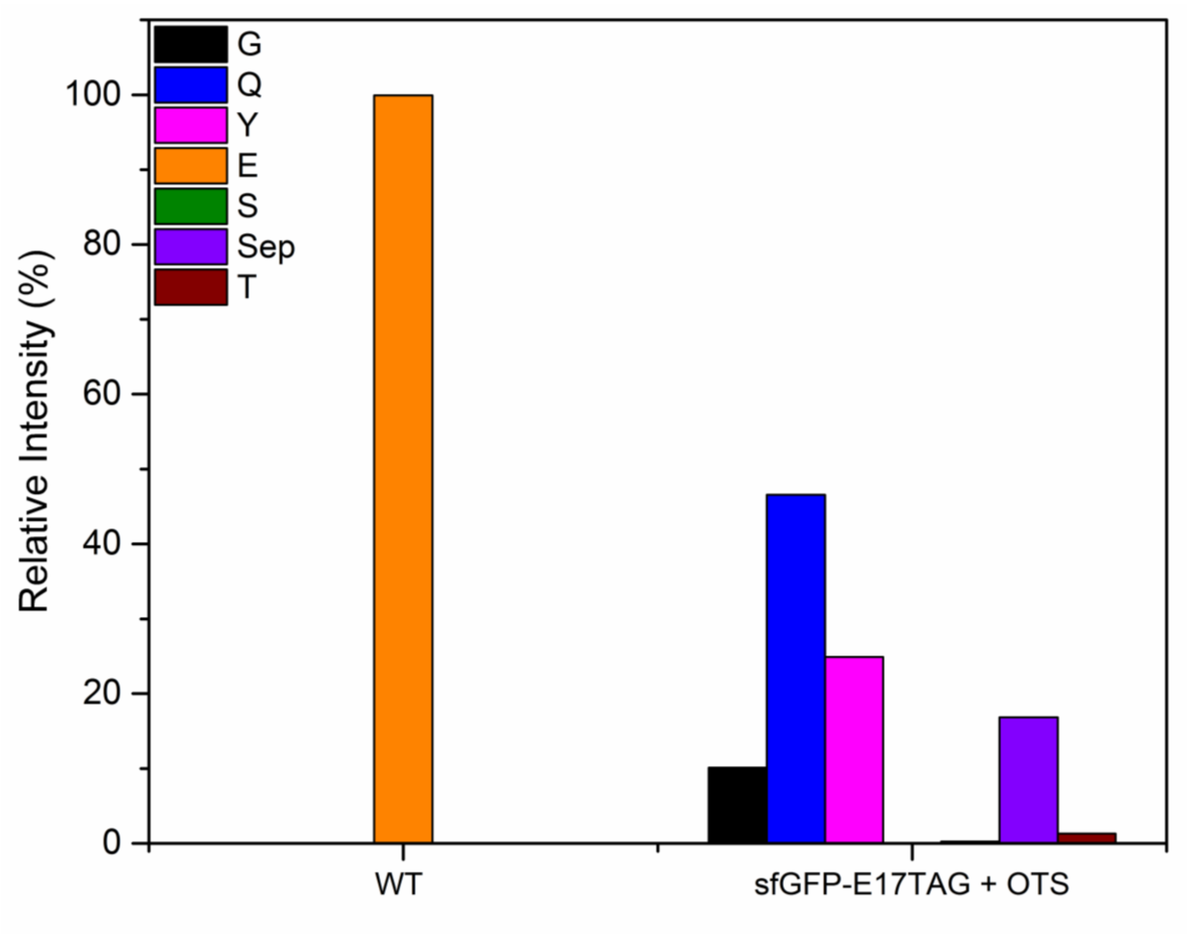

Supplementary Figure 4. Quantitation of Sep incorporation into sfGFP at position E17. The normalized MS1 intensity of peptides reporting incorporation of amino acids are plotted as a percentage of the total ion intensity of peptides reporting incorporation of amino acids at position E17 of sfGFP (see Supplementary Table 3).

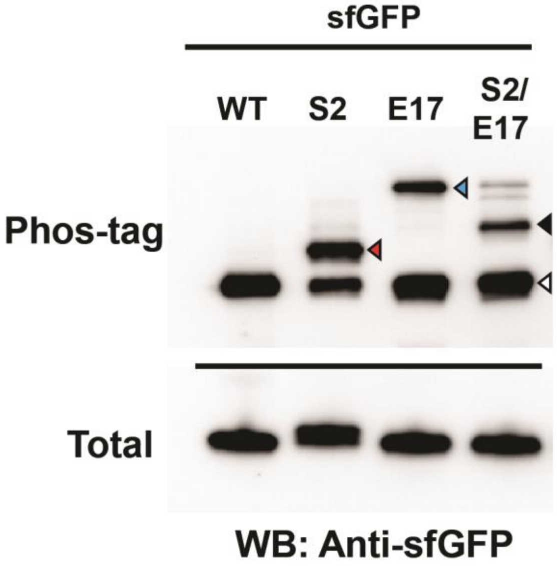

Supplementary Figure 5. Validation of Sep incorporation in sfGFP-S2TAG, sfGFP-E17TAG, and sfGFP-S2TAG/E17TAG by Phos-tag gel Western blot analysis. Unique phospho-sfGFP shifted bands for samples sfGFP-S2TAG (red arrow), sfGFP-E17TAG (blue arrow), sfGFP-S2TAG/E17TAG (black arrow), and unphosphorylated sfGFP are observed (white arrow). Equal amounts of each sfGFP variant were loaded as verified by Western blot analysis probing for total sfGFP (Anti-sfGFP).

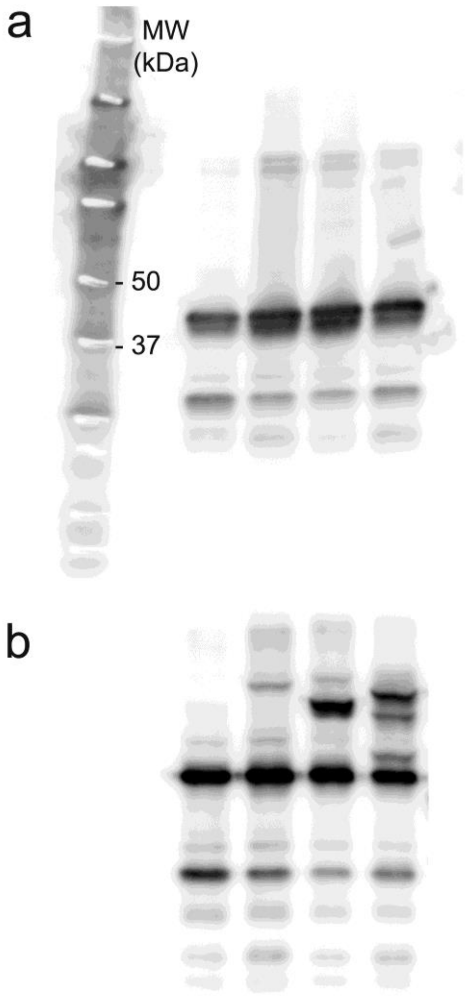

Supplementary Figure 6. Uncropped Western blots for assessing MEK1 expression. (a) Uncropped blot with molecular weight marker showing total MEK1 (from main text Fig. 2c). **(b)** Uncropped blot of the Phos-tag Western blot for phospho-MEK1 showing total MEK1 (from main text Fig. 2c).

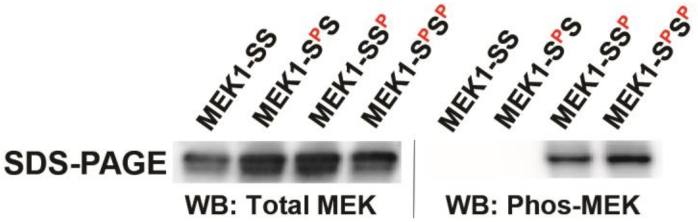

Supplementary Figure 7. Validation of MEK1 biosynthesis via Western blot. Western blot analysis of unphosphorylated MEK1 (MEK1-SS), singly (MEK1-S^PS, MEK1-S^PS), and doubly phosphorylated MEK1 (MEK1-S^PS^P) variants**.** Equal volumes of affinity purified MEK kinases containing activation loop serines (S) or phosphoserines (S^P) at positions 218/222 were probed with an anti-6His antibody for total protein and phospho-specific MEK1 antibody (MEK1-S^PS^P) respectively. Our data indicate that the anti-phos-MEK1 antibody (which binds MEK1-S^PS^P) also binds MEK1-SS^P, but not MEK1-S^PS.

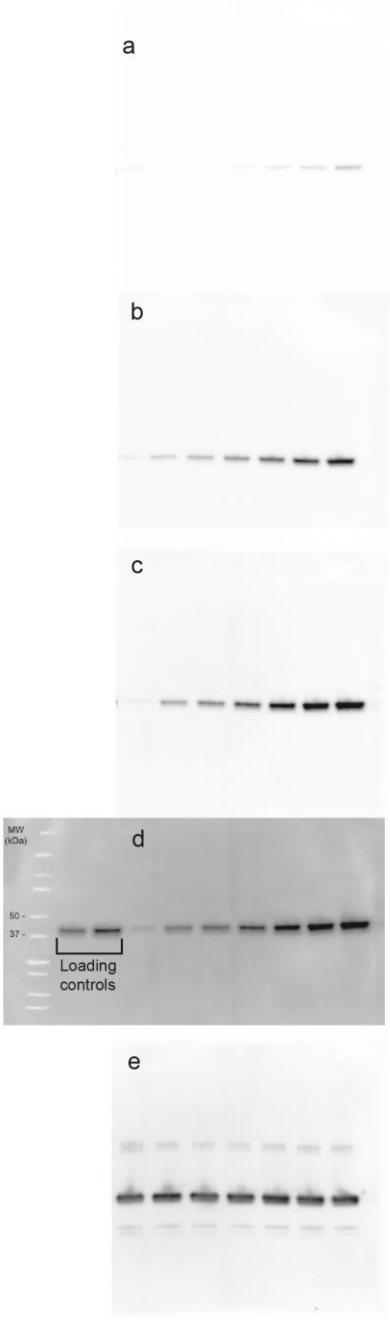

Supplementary Figure 8. Uncropped Western blots for assessing activity of phospho-MEK1 variants (from main text Fig. 3b). Time course study for the production of phospho-ERK upon addition of **(a)** MEK1-SS, **(b)** MEK1-S^PS, **(c)** MEK1-SS^P, and **(d)** MEK1-S^PS^P**.** MEK1 kinase activity was measured at 0.5 min, 1 min, 2 min, 5 min, 10 min, and 30 min time points using kinase dead ERK2 as a substrate. **(e)** Total ERK was also assessed.

Supplementary Tables

Supplementary Table 1: Identification of N-terminal peptides from sfGFPs reporting incorporation of amino acids at positions S2 or E17 of sfGFP-S2TAG and sfGFP-E17TAG. All peptides are reported with a FDR cut-off of 1% and a minimum delta score of 5. Amino acids incorporated at TAG are in red. Peptides reporting skipping at TAG are in bold. Peptides reporting incorporation of K or R are marked with (K/R)* indicating that the amino acid K or R was cleaved off during tryptic digestion. Abbreviations: (de) deamidation, (ox) oxidation, (ph) phosphorylation.

Peptide Sequence	PEP Score	Andromeda Score	MS/MS Count	*m/z*	Mass Error [ppm]
**WT sfGFP**					
_SKGEELFTGVVPILVELDGDVNGHK	6.41E-12	145.6	1	884.8083	-1.2
_SKGEELFTGVVPILVELDGDVNGHK	7.44E-03	61.0	1	531.2834	-0.6
_SKGEELFTGVVPILVELDGDVNGHK	5.30E-12	147.4	1	1326.6976	-1.1
_SKGEELFTGVVPILVELDGDVNGHK	7.58E-08	114.2	3	663.8524	-0.4
_SKGEELFTGVVPILVELDGDVNGHK	1.38E-11	133.8	1	663.8524	-0.2
_SKGEELFTGVVPILVELDGDVNGHK	4.28E-08	120.9	1	884.8008	-0.4
_SKGEELFTGVVPILVELDGDVNGHK	1.65E-05	96.9	1	663.8524	0.0
_SKGEELFTGVVPILVELDGDVN(de)GHK	2.12E-04	81.9	1	531.4802	1.4
**S2=TAG sfGFP+OTS**					
(K/R)*_KGEELFTGVVPILVELDGDVNGHK	1.23E-11	133.1	2	855.7902	-2.1
(K/R)*_KGEELFTGVVPILVELDGDVNGHK	4.49E-03	53.3	1	642.0944	-1.6
_MGKGEELFTGVVPILVELDGDVN(de)GHK	7.60E-13	132.8	1	689.3559	-0.2
_M(ox)GKGEELFTGVVPILVELDGDVNGHK	5.88E-06	99.1	1	923.8091	0.5
**_MKGEELFTGVVPILVELDGDVNGHK**	2.04E-08	125.4	1	899.4703	0.9
**_MKGEELFTGVVPILVELDGDVNGHK**	1.03E-07	108.6	1	674.8546	-0.3
_MQKGEELFTGVVPILVELDGDVNGHK	6.52E-02	39.2	1	942.1565	-0.5
_M(ox)S(ph)KGEELFTGVVPILVELDGDVNGHK	9.61E-07	102.4	1	960.4681	-0.8
_M(ox)S(ph)KGEELFTGVVPILVELDGDVNGHK	2.01E-05	87.7	1	1440.1985	0.4
_M(ox)S(ph)KGEELFTGVVPILVELDGDVNGHK	3.39E-09	115.9	1	960.4681	-1.2
_M(ox)S(ph)KGEELFTGVVPILVELDGDVNGHK	7.58E-03	60.7	1	720.6029	3.6
_MS(ph)KGEELFTGVVPILVELDGDVNGHK	1.28E-13	142.9	1	955.1364	-0.7
_MS(ph)KGEELFTGVVPILVELDGDVNGHK	5.67E-09	106.6	1	716.6041	-0.6
_MS(ph)KGEELFTGVVPILVELDGDVNGHK	2.15E-09	120.8	3	716.6041	0.3
_MS(ph)KGEELFTGVVPILVELDGDVNGHK	5.34E-06	99.0	3	955.1364	-1.5
_MS(ph)KGEELFTGVVPILVELDGDVNGHK	2.20E-05	86.2	1	716.6041	0.3
_MS(ph)KGEELFTGVVPILVELDGDVNGHK	5.56E-09	107.0	1	955.1364	-2.6
_MS(ph)KGEELFTGVVPILVELDGDVNGHK	1.18E-02	51.6	1	955.1364	0.2

Peptide Sequence	PEP Score	Andromeda Score	MS/MS Count	*m/z*	Mass Error [ppm]
_MS(ph)KGEELFTGVVPILVELDGDVNGHK	3.10E-03	70.2	1	955.1364	0.8
_MS(ph)KGEELFTGVVPILVELDGDVNGHK	3.41E-03	69.5	1	955.1364	0.2
_MS(ph)KGEELFTGVVPILVELDGDVNGHK	5.90E-03	64.2	1	955.1364	0.3
_M(ox)S(ph)KGEELFTGVVPILVELDGDVN(de)GHK	1.53E-05	88.5	1	960.7961	0.0
_MS(ph)KGEELFTGVVPILVELDGDVN(de)GHK	4.37E-05	83.0	1	1432.6930	0.8
_SKGEELFTGVVPILVELDGDVNGHK	2.52E-05	93.6	1	884.8008	-2.2
_S(ph)KGEELFTGVVPILVELDGDVN(de)GHK	2.25E-07	105.5	1	1367.1728	0.4
_S(ph)KGEELFTGVVPILVELDGDVNGHK	6.48E-08	117.9	3	911.4563	-1.2
_S(ph)KGEELFTGVVPILVELDGDVNGHK	5.54E-08	119.6	1	911.4563	0.4
_S(ph)KGEELFTGVVPILVELDGDVNGHK	1.04E-07	111.0	2	911.4563	0.7
_SKGEELFT(ph)GVVPILVELDGDVNGHK	3.50E-02	52.9	1	911.4563	0.8
**S2=TAG sfGFP-OTS**					
_KGEELFTGVVPILVELDGDVNGHK	5.43E-02	15.9	1	642.0944	-0.7
_KGEELFTGVVPILVELDGDVNGHK	4.50E-04	74.6	2	642.0944	-1.5
_KGEELFTGVVPILVELDGDVNGHK	3.63E-17	162.7	1	1283.1816	0.3
_KGEELFTGVVPILVELDGDVNGHK	3.13E-05	84.7	1	855.7902	-1.7
_KGEELFTGVVPILVELDGDVNGHK	5.83E-02	9.5	1	642.0944	0.4
_KGEELFTGVVPILVELDGDVNGHK	2.53E-05	88.2	1	855.7902	0.3
**_MKGEELFTGVVPILVELDGDVNGHK**	2.88E-05	92.3	1	674.8546	-1.2
**_MKGEELFTGVVPILVELDGDVNGHK**	4.42E-06	101.5	1	899.4703	-0.4
_M(ox)KGEELFTGVVPILVELDGDVNGHK	1.08E-05	99.9	2	904.8020	-0.9
_MQKGEELFTGVVPILVELDGDVNGHK	1.92E-13	134.0	1	706.8692	-1.4
_MQKGEELFTGVVPILVELDGDVNGHK	1.46E-09	122.8	1	942.1565	-0.5
_MQKGEELFTGVVPILVELDGDVNGHK	1.89E-09	120.9	1	706.8692	0.1
_M(ox)YKGEELFTGVVPILVELDGDVN(de)GHK	8.41E-06	95.1	1	719.8651	0.1
_M(ox)YKGEELFTGVVPILVELDGDVNGHK	3.10E-05	81.8	1	959.1564	-0.1
_M(ox)YKGEELFTGVVPILVELDGDVNGHK	3.26E-03	70.6	1	719.6191	-1.1
**E17=TAG sfGFP+OTS**					
_GEELFTGVVPILVGLDGDVNGHK	5.80E-24	145.0	2	824.4321	-1.5
_GEELFTGVVPILVK	9.92E-04	55.7	3	750.9372	-0.6
_GEELFTGVVPILVQLDGDVNGHK	1.21E-07	74.4	3	1218.6421	-1.6
_GEELFTGVVPILVQLDGDVN(de)GHK	1.56E-05	75.8	2	610.0707	2.7
_GEELFTGVVPILVS(ph)LDGDVNGHK	4.38E-03	50.0	1	825.7438	-0.6
_GEELFTGVVPILVTLDGDVNGHK	9.34E-05	49.1	2	803.7602	-0.7
_GEELFTGVVPILVYLDGDVNGHK	5.02E-21	130.4	2	1236.1445	-1.4
(K/R)* _LDGDVNGHK	2.05E-05	131.4	2	477.7356	-0.3
_SKGEELFTGVVPILVGLDGDVNGHK	4.08E-14	117.6	2	1290.6870	-1.8
_SKGEELFTGVVPILVK	1.87E-15	156.2	5	858.5007	-0.2
_SKGEELFTGVVPILVQLDGDVNGHK	3.15E-34	176.9	8	884.4728	-0.6

Peptide Sequence	PEP Score	Andromeda Score	MS/MS Count	*m/z*	Mass Error [ppm]
_SKGEELFTGVVPILVS(ph)LDGDVNGHK	2.56E-04	100.7	3	1345.6755	0.5
_SKGEELFTGVVPILVSLDGDVNGHK	2.41E-02	43.2	1	870.7973	-2.7
_SKGEELFTGVVPILVYLDGDVNGHK	1.22E-02	47.8	2	896.1411	-1.3

Supplementary Table 2: Label free quantitation of amino acid incorporation at position S2 of sfGFP by spectral counting. The number of peptide identifications for each amino acid is reported (- OTS, incorporation in the absence of the Sep-OTS; + OTS, incorporation in the presence of the Sep-OTS).

Sample	G	Skip (M)	Q	Y	S	K or R	Sep
WT sfGFP	0	0	0	0	8	0	0
sfGFP-S2TAG - OTS	0	3	3	3	0	6	0
sfGFP-S2TAG + OTS	2	2	1	1	2	2	20

Supplementary Table 3: Label-free MS1 intensity based quantitation of amino acid incorporation at position S2 and E17 of sfGFP. The relative intensity of all detected peptides reporting the incorporation of Sep and natural amino acids is reported relative to the sum of all intensities of peptides reporting incorporation of amino acids at position S2 of sfGFP (- OTS, incorporation in the absence of the Sep-OTS; + OTS, incorporation in the presence of the Sep-OTS). Peptides reporting incorporation of K in sfGFP-E17TAG + OTS were excluded for the quantitation of the E17 reporter as these peptides likely have significantly altered ionization properties compared with the full-length peptide.

Sample	G	Skip (M)	Q	Y	S	K or R	Sep	Thr
WT sfGFP					100.0			
sfGFP-S2TAG - OTS		12.2	15.71	41.42		30.64		
sfGFP-S2TAG + OTS	0.5	2.1	1.3		1.6	7.1	87.4	
sfGFP-E17TAG + OTS	10.1		46.6	24.9	0.2	-*	16.8	1.3

* K containing peptides were excluded from analysis.

Supplementary Table 4: Primer sequences used in cloning (see *Supplementary Methods*)**.**

Primer ID	Sequence (5’ to 3’)
P1	TAGAAAGGTGAAGAACTGTTTAC
P2	CATATGTATATCTCCTTCTTAAAGTTAAAC
P3	GTGCCGATTCTGGTGTAGCTGGATGGCGATGTG
P4	CACATCGCCATCCAGCTACACCAGAATCGGCAC
P5	CGACTAGATGGCTAACTAGTTCGTTGGCACCCGTTC
P6	GAACGGGTGCCAACGAACTAGTTAGCCATCTAGTCG
P7	GACTAGATGGCTAACTCTTTCGTTGGCACCCGTTC
P8	GAACGGGTGCCAACGAAAGAGTTAGCCATCTAGTC
P9	GGTCAGCTGATCGACTCTATGGCTAACTAGTTCG
P10	CGAACTAGTTAGCCATAGAGTCGATCAGCTGACC
P11	CAAAGTTCGAGTAGCTATCCGTAAAATCAGCCCCTTTGAG
P12	CTCAAAGGGGCTGATTTTACGGATAGCTACTCGAACTTTG
P13	TGGAGCCATCCGCAGTTCGAAAAATAAGGCGCGCCGGATCCGAATTCG
P14	CGCAGCTGCGGCTGCCAC

Supplementary Methods

Cell growth and harvesting

Starter cultures of *E. coli* C321.ΔA cells[1] in LB media were grown overnight at 30°C and then diluted to a starting OD_{600} of 0.05AU in 1L sterile 2xYTPG media prepared in 2.5L Tunair flasks (IBi Scientific, Peosta, IA). C321.ΔA was derived from a previously described strain[1] with the following genotype modifications: ΔmutS:zeo, ΔtolC, Δbla:tolC, SerB-/ΔSerB. SerB is a phosphoserine phosphatase, and deletion is required for sufficient levels of Sep in the cytoplasm for protein synthesis[2]. The 2xYTPG media consisted of 16g/L tryptone, 10g/L yeast extract, 5g/L NaCl, 7g/L K_2HPO_4, 3g/L KH_2PO_4 and 18g/L glucose. The pH value of 2xYTPG media was adjusted to a pH value of 7.1 with KOH. Cultures expressing the SepOTS were supplemented with 2mM L-phosphoserine and the SepOTS system was induced at an OD_{600} of 0.6AU with 1mM IPTG. Cultures were grown to a final OD_{600} of 3.0AU in an Innova 44 shaker incubator (New Brunswik Scientific, Enfield, CT) operated at 30°C with vigorous shaking at 220 RPM. Cells were pelleted at 4°C and washed three times with ice-cold S30 buffer (10mM Tris-acetate pH 8.2, 14mM magnesium acetate, 60mM potassium acetate, 1mM dithiothreitol). Bacterial cell pellets were stored at -80°C until extract preparation.

CFPS extract preparation

Cell pellets were thawed on ice and resuspended in ice cold S30 buffer using a cell weight to buffer ratio of 1.0g wet cell mass to 0.8ml S30 buffer. Cells were lysed on ice by sonication with a model Q125 sonicator (QSonica LLC, Newtown, CT). The lysis program consisted of three 45s bursts performed at 50% amplitude and 59s pause between subsequent bursts. The total deposited energy of approximately 900 Joules[3]. Lysate was supplemented with an additional 2mM Dithiothreitol (DTT) and cell debris was removed with a 10min spin performed at 12,000 x g at 4°C. The clarified cell extract was incubated for 1h at 37°C while shaking at 220 RPM and insoluble material was removed by centrifugation performed at 12,000 x g for 30min at 4°C. The supernatant was flash-frozen in liquid nitrogen and stored at -80°C until use for CFPS.

Protein quantification of sfGFP and MEK1

Expression of active sfGFP proteins in CFPS extracts was quantified by fluorescence spectroscopy. Briefly, CFPS reactions were diluted 50x with 50mM HEPES buffer pH 7.5, transferred into Costar model #3694 (Corning Incorporated, Corning, NY) 96-well flat bottom half area black plates and fluorescence emission was measured on a Synergy 2 (BioTek

Instruments Incorporated, Winooski, VT) plate reader fitted with a 510nm cutoff filter. The excitation and emission wavelength were 485nm and 528nm respectively. The sfGFP concentration was calculated using a standard curve established with ^{14}C-leucine quantified sfGFP as described previously[5]. For time-course analysis of protein synthesis, reactions were scaled to 30µl volumes and incubated at 30°C in a Synergy 2 (BioTek Instruments Incorporated, Winooski, VT) plate reader while fluorescence was monitored every 5 minutes for 20 hours in 96-well flat bottom half area black plates.

Total MEK1 protein production was quantified by monitoring the incorporation of radioactive ^{14}C-leucine into proteins following a protocol by Swartz and colleagues[5,6]. Briefly, CFPS reactions were split evenly and deposited onto two 3 MM filter papers (Whatman) and either washed with 5% trichloroacetic acidic acid or dried without washing (untreated sample). Filters were dried for 1h under a heat lamp and radioactivity was measured by liquid scintillation counting on a MicroBeta2 instrument (PerkinElmer, Waltham, MA) and reported as counts per minute (cpm). The molecular weight and number of leucine residues in MEK1 was calculated with ProtParam software[7] (http://web.expasy.org/protparam/). The volumetric yield of protein was then calculated using the following equation: [((Washed cpm - Background cpm) / Unwashed cpm) * [Leu in reaction] µM * (MW Protein µg/µmol)] / [(# Leu residues) * (1000 mL/L)] = µg/mL protein produced.

Protein purification

For C-terminal Strep-tagged sfGFP purification, CFPS reactions were applied to Strep-tactin Sepharose mini-columns (IBA GmbH, Gottingen, Germany) and proteins were purified according to the instructions provided with the mini-columns. Purified protein was then concentrated and buffer exchanged into 10mM Tris-HCl buffer pH 7.4 using 10,000 MWCO Amicon (Millipore, Billerica, MA) centrifugal filters. The total protein concentration was estimated by fluorescence spectroscopy with a GFP standard curve as described above. The concentration of sfGFP was further verified by densitometry performed on coomassie stained SDS-PAGE gels using a BSA dilution series run on the same gel as a reference.

Purification of MEK1 proteins from CFPS reactions was performed with Ni-affinity chromatography. CFPS reactions (~100µl) were first diluted with 600µl denaturing purification buffer (20mM sodium phosphate pH 7.4, 500mM NaCl, 8M urea) and combined with 100µl bed volume of Ni-Agarose beads (Qiagen, Valencia, CA). Proteins were incubated with the beads

for 1h at 4°C while rotating on an end-over-end tube rotator. The beads were then washed 3x with 1ml denaturing purification buffer. Proteins were eluted 3 times with 200µl elution buffer (20mM sodium phosphate pH 7.4, 500mM NaCl, 8M urea, 250mM imidazole,). Elution was performed for 5min at 4°C on an end-over-end tube rotator. The combined protein fractions were combined and buffer exchanged with 10mM Tris-HCl buffer pH 7.4 with 10kDa MWCO devices as described above. Note that half of the MEK1-S^PS^P sample was buffer exchanged with 10mM Tris-HCl buffer pH 7.4. The remainder was exchanged into cold storage buffer (50mM Tris-HCl pH 7.4, 150mM NaCl, 20% glycerol, 1mM DTT). The protein concentration of buffer exchanged samples was determined by UV absorbance spectroscopy at 280nm on a nanodrop (Thermo Scientific, Wilmington, DE).

Expression of kinase inactive human ERK2 (K54R)

BL21(DE3) cells were transformed with the pET15-ERK2 plasmid of kinase inactive human ERK2 (K54R) and the culture was grown at 37°C, 230 RPM to an OD_{600} of 0.8AU in LB media supplemented with 0.08% glucose, and 100µg/ml ampicillin. Protein expression was then induced with 1mM IPTG, and protein expression proceeded for ~20h at 20°C and 230 RPM. Cells were pelleted (10 min at 4000 x g at 4°C) and the cell pellet was frozen for at least 1 h at -80°C. The cell pellet was briefly thawed in a 37°C water bath followed by resuspension in 5ml of ice-cold bacterial lysis buffer (50mM Tris-HCl pH 7.4, 500mM NaCl, 0.5mM EDTA, 0.5mM EGTA, 1mM DTT, 1mg/ml lysozyme, 50mM NaF, 1mM $NaVO_4$, 10% glycerol, complete mini-EDTA-free protease inhibitor cocktail tablets) and incubated on ice for 30min, followed by sonication. The lysates were centrifuged at 22,000 x g for 15min at 4°C. The clarified lysate was purified by Ni-affinity chromatography with 200µl Ni-NTA agarose resin (Qiagen Valencia, CA) using Pierce spin columns (Thermo Scientific Waltham, MA). The resin was equilibrated with 5ml of Ni-NTA equilibration buffer (50mM Tris-HCl pH=7.4, 500mM NaCl, 0.5mM EDTA, 0.5mM EGTA, 1mM DTT, 50mM NaF, 1mM $NaVO_4$, 10% glycerol). The clarified lysate was loaded onto the column via syringe. The column was then washed once with 10ml of Ni-NTA wash buffer (50mM Tris-HCl pH=7.4, 500mM NaCl, 0.5mM EDTA, 0.5mM EGTA, 1mM DTT, 50mM NaF, 1mM $NaVO_4$, 10% glycerol, 20mM imidazole). Protein was eluted using Ni-NTA elution buffer (50mM Tris-HCl pH 7.4, 500mM NaCl, 0.5mM EDTA, 0.5mM EGTA, 1mM DTT, 50mM NaF, 1mM $NaVO_4$, 10% glycerol, 250mM imidazole) and 400µl elutions were collected in 1.5ml Eppendorf tubes. Eluted protein fractions were analyzed by SDS-PAGE and fractions containing the target protein were pooled and buffer-exchanged into the protein storage buffer (50mM Tris-HCl pH=7.4, 150mM NaCl, 1mM DTT, 20% glycerol) using 0.5 ml 10kDa MWCO ultra

centrifugal filters Amicon (Millipore Billerica, MA) and the protein was stored at −20°C. The protein concentration was quantified by UV absorbance spectroscopy at 280nm and by comparing known quantities of BSA standards (Pierce, Rockford, IL) on a coomassie stained SDS-PAGE gel.

Cloning

pY71-sfGFP was previously described[4]. S2TAG sfGFP was constructed from pY71-sfGFP using inverse PCR with Primers 1 & 2 (Supplementary Table 4). The E17TAG mutations were made in the pY71-sfGFP and pY71-S2TAG sfGFP by site-directed mutagenesis using Primers 3 & 4 (Supplementary Table 4).

The gene for MBP-MEK1 S218/S222 was digested from the previously described pCG-MBP-MEK1 SS plasmid[2] using XbaI blunt/HindIII restriction enzymes and subcloned into pCRT7 NT Topo tetR pLtetO that had been digested with KpnI blunt /HindIII restriction enzymes. Blunting was performed using quick blunting enzyme (New England Biolabs). The gene for the MBP-MEK1 S218TAG/S222E was subcloned from the previously reported pCG-MBPMEK1XE plasmid[2] using XbaI/HindIII restrictions sites. The fragment containing MBP-MEK1 was blunted and treated with calf intestinal alkaline phosphatase (CIP, NEB). The fragment between the KpnI/HindIII sites in pCRT7 NT Topo tetR pLtetO was removed and the restriction sites were blunted. The MBP-MEK1 S218TAG/S222E fragment was ligated into the blunted KpnI/HindIII sites of the pCRT7 NT Topo tetR pLtetO plasmid. S222E was then mutated to TAG by site-directed mutagenesis using primers P5 & P6 to create MBP-MEK1 S218TAG/S222TAG. For the experiments described here, the MEK1 S218/S222 and S218TAG/S222TAG genes were further subcloned by digesting both MEK1 fragment with NdeI-SalI cloning between the same restriction sites in the previously described pY71 plasmid[4]. MEK1 S218TAG/S222 and S218/S222TAG were created from MEK1 S218TAG/S222TAG using site-directed mutagenesis with primers P7 & P8 and P9 & P10, respectively. We also constructed Strep-tag MEK1 variants. For these, the C-terminal 6xHis-tag for MEK1 S218/S222, MEK1 S218/S222TAG, and MEK1 S218TAG/S222TAG was replaced with a C-terminal Strep-tag via inverse PCR using primers P13 & P14. The previously described pET15-ERK2 plasmid[2] was used to construct a K54R mutation in ERK2 which inactivates the kinase. Lys54 was mutated by site-directed mutagenesis using primers P11 & P12. The construction of the SepOTS plasmid (OTS1.5) was previously described by Aerni[8].

Protein digestion for mass spectrometry

Digestion of sfGFPs: Affinity purified and buffer exchanged protein was digested with trypsin according to an optimized protocol for in solution digestion of sfGFP reported by Aerni[8]. Briefly, an estimated 5µg of the protein was dissolved in 10µl digestion buffer consisting of 0.5% acid labile surfactant (ALS-110, Protea), 10mM DTT and 1mM EDTA in 10mM Tris-HCl pH=8.0 (23 °C). The reaction was incubated for 35 min at 55 °C in a water bath and then quenched for 30s on ice. Then 4µl of an aqueous 60mM iodoacetamide (IAA) solution was added and alkylation of cysteines proceeded for 30 min at room temperature and in the dark. Excess IAA was quenched with 3.7µl of a 25mM DTT solution. Finally 10µl of 1M Tris-HCl buffer pH=8.0 and 77.5µl of 70mM Tris-HCl pH=8.0 containing 2mM $CaCl_2$ were added followed by sequencing grade trypsin prepared at 0.5µg/µl. The amount of sequencing grade porcine trypsin was adjusted to obtain a protein/trypsin ratio of 15:1 by weight. Acid hydrolyzed digests were desalted using StageTips as described by Rappsilber[9] with custom-built stage tips. The tips consisted of two 1.06mm diameter punches of Empore C_{18} product #2215 (3M, St. Paul, MN) fitted into a 200µl HydroLogix pipette tip (Molecular BioProducts Inc., San Diego, CA). Each sample was desalted sequentially with 2 StageTips to ensure complete peptide recovery. Peptides were eluted with 50µl of 0.1% trifluoroacetic acid (TFA) in 80% acetonitrile. Peptides were dried for 30min in a rotary vacuum centrifuge operated at room temperature. Dried peptides were reconstituted in 11µl resuspension buffer consisting of 30:18:52 by volume 70% formic acid, 1-propanol and 0.1% formic acid (FA). The concentration of peptides was determined by UV_{280} on a nanodrop UV spectrometer (Thermo Fisher Scientific, Waltham, MA). The peptide concentration range was between 0.5-1.1µg/µl across all samples analyzed. Peptide stock solutions were then diluted to a concentration of 60ng/µl using 0.1% FA and 300ng peptides were injected for LC-MS/MS analysis.

Digestion of MEK1 protein samples

The following MEK1 constructs were analyzed by mass spectrometry: MEK1 SS (S218S/S222S) and MEK1 S^pS^p (S218TAG/S222TAG). Affinity purified MEK1 protein was prepared in 10mM Tris-HCl buffer pH=7.4 and quantified by UV_{280} spectroscopy as described above. Aliquots corresponding to 5µg total protein were flash-frozen and then dried in a vacuum centrifuge operated without heat at a pressure of 0.3mbar. The dried protein was dissolved in 12.5µl solubilization buffer consisting of 0.5% w/v acid labile surfactant II (Protea Biosciences, Morgantown, WV), 10mM DTT and 1mM EDTA in 50mM Tris-HCl buffer pH=8.0 and samples were incubated for 35min at 35°C in a water bath. Reactions were quenched on ice and 2µl 1M

Tris-HCl buffer pH=8.0 and 4.67µl freshly prepared 100mM aqueous iodoacetamide (IAA) (Sigma Aldrich, St. Louis, MO) solution was added. Samples were incubated for 30min at room temperature and in the dark. Excess IAA in the reaction was quenched by adding 0.7µl of 200mM DTT. Protein was digested with Lysyl Endopeptidase (LysC) (Wako Chemicals, Richmond, VA) and sequencing grade trypsin (Promega, Madison, WI). Digestion with LysC proceeded for 4h at 37°C with 0.0007AU LysC per digest. Digest were then diluted with 113.3µl water, 0.667µl of 1M $CaCl_2$ and 13.33µl of 1M Tris-HCl buffer pH=8.0. Digestion with trypsin was performed with 0.9µg sequencing grade trypsin resulting in a trypsin/protein ratio of ~1:5.6. Digests were then acidified (pH<2.0) with 12.5µl 20% TFA solution and acid cleavable detergent was hydrolyzed for 15min at 23°C. Digests were desalted with C_{18} StageTips as described for the sfGFP digests. Finally, dried peptides were reconstituted in 10µl 50% acetonitrile with 0.1% formic acid, quantified by UV_{280} and 500ng peptides were transferred into conical high recovery HPLC glass vials (Agilent, Santa Clara, CA). Solvent was removed with a gentle stream of nitrogen gas and dried peptides were reconstituted in 10µl LC-MS solvent consisting of 5% DMSO, 5mM sodium phosphate prepared in 3:8 by volume 70% formic acid and 0.1% TFA. A total of 3µl corresponding to 150ng peptides were injected for LC-MS/MS analysis. MASCOT searches identified significant peptides spanning 45% of the MEK1 SS (S218S/S222S) protein sequence and 41% of the MEK1 S^pS^p (S218TAG/S222TAG) sequence. The activation loop segment containing serines S218S/S222S in MEK1 SS was identified ($[M+2H]^{2+}$=1187.5621^{+2} Da^{+2}; L<u>C</u>DFGVSGQLID**S**MAN**S**FVGTR; **S218S/S222S;** <u>C</u> Carbamidomethyl; MASCOT score 121) while neither this peptide, nor a modified peptide, was observed in MEK1 S^pS^p (S218TAG/S222TAG). We conclude that the missing phosphopeptide data results from the addition of multiple phosphorylation sites severely compromising the ionization efficiency of the phosphopeptide or the two negative charges (S^pS^p) on the peptide shifts the charge state to a singly charged peptide and places the observable *m/z* outside of our detection window.

Mass spectrometry

Nanoflow LC-MS/MS analysis peptides was performed on a Orbitrap Velos[10] mass spectrometer (Thermo Scientific, Waltham, MA) operated in data dependent acquisition mode with a top10 HCD method. Details of the mass spectrometry method and the nano-LC setup were described previously by Aerni[8]. sfGFP digests were analyzed with the following gradient program in place of the previously reported 90 min gradient: (min/%B) 0.0/2.0, 0.1/2.0, 2.0/10.0, 60.0/25.0, 70.0/40.0, 72.0/95.0, 78.0/95.0, 80.0/2.0, 90.0/2. MEK1 samples were analyzed with

the following 200 min gradient program: (min/%B) 0.0/2.0, 0.1/2.0, 2.0/7.0, 166.0/30.0, 176.0/45.0, 177.0/95.0, 182.0/95.0, 200.0/2.0.

Bioinformatics

Tandem MS data from the analysis of sfGFP samples were searched with MaxQuant[11] version 1.4.1.2 using the default search parameters unless noted otherwise. Spectra were matched against the *E. coli* database EcoCyc[12] v.17 and against a custom sfGFP databases for identification of amino acid incorporation at position S2=TAG and positions E17=TAG of the sfGFP reporter. The custom protein database contained 20 variants of the sfGFP protein sequence considering any of the 20 natural amino acids at the position of the TAG codon. A detailed description of this workflow for a similar GFP reporter has been recently reported[8]. The amino acid sequence of the sfGFP reporter used here was as follows:

MXKGEELFTGVVPILVXLDGDVNGHKFSVSGEGEGDATYGKLTLKFICTTGKLPVPWPTLVTTF
SYGVQCFSRYPDHMKRHDFFKSAMPEGYVQERTISFKDDGNYKTRAEVKFEGDTLVNRIELKG
IDFKEDGNILGHKLEYNYNSHNVYITADKQKNGIKANFKIRHNIEDGSVQLADHYQQNTPIGDGP
VLLPDNHYLSTQSALSKDPNEKRDHMVLLEFVTAAGITHGMDELYKLEHHHHHH

Note that the position of the stop codons at position S2 and E17 are marked with "**X**". MaxQuant was configured with the fixed modification carbamidomethyl (C) and variable modifications oxidation (M) and phosphorylation (STY) respectively. The enzyme specificity was trypsin/P considering only fully tryptic peptides with up to 3 missed cleavages. All searches were performed with a precursor and fragment ion mass tolerance of 4.5ppm and 20 ppm respectively. Peptide and protein identifications were reported with an estimated false discovery rate (FDR) of 1% and identifications matching the reverse database were filtered out with Perseus[13] software (v. 1.4.0.20). Only peptides that had a minimum delta score >5 were reported.

Database searching of MEK1 MS data was performed with MaxQuant[11] version 1.5.1.2. Unless noted otherwise, default search parameters were used. The enzyme specificity was trypsin/P and only fully tryptic peptides with up to 3 missed cleavages were considered for peptide identification. The following variable modifications were specified: Oxidation (M) deamidation (N/Q) carbamidomethyl (C) and carbamylation (N-term, K, R, C, M, S, T, Y). The precursor and fragment ion mass tolerance was 4.5 and 20 ppm respectively and the false discovery rate for reported protein and peptide identifications was set to 1%. Reverse database hits and peptides

matching common contaminants were removed in Microsoft Excel. Spectra were matched against the *E. coli* protein database EcoCyc10 v.17 and a custom database considering 64 MEK protein sequences. The MEK database contained all combinations of the natural amino acids S, Q, K, Y, G, T, V and R at any of the 2 TAG codon sites. The database consisted of the following protein sequence for MEK1:

MPKKKPTPIQLNPAPDGSAVNGTSSAETNLEALQKKLEELELDEQQRKRLEAFLTQKQKVGEL
KDDDFEKISELGAGNGGVVFKVSHKPSGLVMARKLIHLEIKPAIRNQIIRELQVLHECNSPYIVGF
YGAFYSDGEISICMEHMDGGSLDQVLKKAGRIPEQILGKVSIAVIKGLTYLREKHKIMHRDVKPS
NILVNSRGEIK<u>LCDFGVSGQLIDXMANXFVGTR</u>SYMSPERLQGTHYSVQSDIWSMGLSLVEMA
VGRYPIPPPDAKELELMFGCQVEGDAAETPPRPRTPGRPLSSYGMDSRPPMAIFELLDYIVNEP
PPKLPSGVFSLEFQDFVNKCLIKNPAERADLKQLMVHAFIKRSDAEEVDFAGWLCSTIGLNQPS
TPTHAAGVAAAAAHHHHHH*

The position of amber codons in MEK1 at position 218 and position 223 is marked by "<u>X"</u>. The reporter LCDFGVSGQLIDXMANXFVGTR is underlined.

Label-free quantitation of amino acid incorporation

Label-free quantitation of amino acid incorporation for sfGFP was performed by spectra counting (Supplementary Fig. 2, Supplementary Table 2) and using MS1 intensity based quantitation (Supplementary Table 3). The MS1 peak intensities for each charge state of all peptides reported in Supplementary Table 1 were determined with Skyline software[9] v. 2.5.0.6157 and the sum of all precursor intensities for each peptide was exported into Microsoft Excel 2013 for further processing. The peptide FEGDTLVNR corresponding to amino acid residue 114-122 of the sfGFP reporter was included to normalize ion intensities between GFP samples as reported earlier[2]. To quantitate the incorporation of amino acids at position S2 and E17, it was assumed that the sum of all normalized ion intensities reporting incorporation of amino acids at position S2 or E17 adds up to 100% and that the ionization efficiency for each peptide is the same. Note that peptides reporting incorporation of K in the E17=TAG sfGFP were excluded from the analysis because cleavage with trypsin (cleaving C terminal of K and R) generates shorter peptides with significantly different ionization properties. The incorporation of each detected amino acid was then calculated as a percentage relative to the sum of the total ion intensities of all peptides for S2 sfGFP and for all peptides except peptides reporting incorporation of K at position E17=TAG (Supplementary Table 3, Supplementary Figs. 3 and 4).

Supplementary References

1. Lajoie, M. J. *et al.* Genomically recoded organisms expand biological functions. *Science* **342,** 357–60 (2013).

2. Park, H.-S. *et al.* Expanding the genetic code of Escherichia coli with phosphoserine. *Science* **333,** 1151–4 (2011).

3. Kwon, Y.-C. & Jewett, M. C. High-throughput preparation methods of crude extract for robust cell-free protein synthesis. *Sci. Rep.* **5,** 8663 (2015).

4. Hong, S. H. *et al.* Cell-free Protein Synthesis from a Release Factor 1 Deficient Escherichia coli Activates Efficient and Multiple Site-specific Nonstandard Amino Acid Incorporation. *ACS Synth. Biol.* (2014). doi:10.1021/sb400140t

5. Swartz, J. R., Jewett, M. C. & Woodrow, K. A. Cell-free protein synthesis with prokaryotic combined transcription-translation. *Methods Mol. Biol.* **267,** 169–82 (2004).

6. Jewett, M. C. & Swartz, J. R. Substrate replenishment extends protein synthesis with an in vitro translation system designed to mimic the cytoplasm. *Biotechnol. Bioeng.* **87,** 465–72 (2004).

7. Wilkins, M. R. *et al.* Protein identification and analysis tools in the ExPASy server. *Methods Mol. Biol.* **112,** 531–52 (1999).

8. Aerni, H. R., Shifman, M. A., Rogulina, S., O'Donoghue, P. & Rinehart, J. Revealing the amino acid composition of proteins within an expanded genetic code. *Nucleic Acids Res.* **43,** e8 (2015).

9. Rappsilber, J., Mann, M. & Ishihama, Y. Protocol for micro-purification, enrichment, pre-fractionation and storage of peptides for proteomics using StageTips. *Nat. Protoc.* **2,** 1896–906 (2007).

10. Olsen, J. V *et al.* A dual pressure linear ion trap Orbitrap instrument with very high sequencing speed. *Mol. Cell. Proteomics* **8,** 2759–69 (2009).

11. Cox, J. & Mann, M. MaxQuant enables high peptide identification rates, individualized p.p.b.-range mass accuracies and proteome-wide protein quantification. *Nat. Biotechnol.* **26,** 1367–72 (2008).

12. Keseler, I. M. *et al.* EcoCyc: fusing model organism databases with systems biology. *Nucleic Acids Res.* **41,** D605–12 (2013).

13. Cox, J. & Mann, M. 1D and 2D annotation enrichment: a statistical method integrating quantitative proteomics with complementary high-throughput data. *BMC Bioinformatics* **13 Suppl 1,** S12 (2012).

14. MacLean, B. *et al.* Skyline: an open source document editor for creating and analyzing targeted proteomics experiments. *Bioinformatics* **26,** 966–8 (2010).
